# Association between prognostic nutritional index and complications in children with immunoglobulin A vasculitis

**DOI:** 10.3389/fnut.2026.1853071

**Published:** 2026-08-17

**Authors:** Xiao Xiong, Zezheng Liu, Huan Li, Yinfei Zhou, Li Yu

**Affiliations:** Department of Pediatrics, Xiangtan Central Hospital Affiliated to Hunan University, Xiangtan, China

**Keywords:** abdominal pain, bloody stool, immunoglobulin A vasculitis, prognostic nutritional index, proteinuria, vomiting

## Abstract

**Objective:**

This study aimed to evaluate the correlation between prognostic nutritional index (PNI) and complications in immunoglobulin A vasculitis (IgAV) children, including vomiting, abdominal pain, bloody stools, and proteinuria.

**Methods:**

A retrospective study was conducted on 732 children diagnosed with IgAV at the Xiangtan Central Hospital of the Hunan University between 2014 and 2025. Logistic regression analyses, subgroup analysis, sensitivity analysis, and receiver operating characteristic (ROC) curves were employed to evaluate the association between PNI and vomiting, abdominal pain, bloody stool, and proteinuria.

**Results:**

Multivariate logistic regression analysis indicated that, each unit increase and one standard deviation (SD) increase in the PNI was associated with a 9.4% reduction (OR = 0.906) and a 50.5% reduction (OR = 0.495) in the risk of vomiting, respectively. Compared to the low PNI group, the high PNI group exhibited a 55.4% reduction in the risk of vomiting (OR = 0.446). Additionally, each unit increase and one SD increase in PNI were linked to a 6.7% (OR = 0.933) and a 38.9% (OR = 0.611) reduction in the risk of abdominal pain, respectively. Furthermore, the high PNI group demonstrated a 60.4% reduction in the risk of abdominal pain (OR = 0.396). For each unit increase and one SD increase in PNI, the risk of bloody stools decreased by 4.9% (OR = 0.951) and 30.3% (OR = 0.697), respectively. Moreover, the high PNI group exhibited a 52.2% reduction in the risk of bloody stools (OR = 0.478). Additionally, for each unit increase and one SD increase in PNI, the risk of proteinuria decreased by 6.6% (OR = 0.934) and 38.5% (OR = 0.615), respectively. Compared to the low PNI group, the high PNI group exhibited a 47.0% reduction in the risk of proteinuria (OR = 0.530). And Most subgroup and sensitivity analyses confirmed the stability of the results (*p* < 0.05). ROC analysis indicated that PNI was significantly correlated with vomiting, abdominal pain, bloody stools, and proteinuria (*p* < 0.05).

**Conclusion:**

Higher levels of PNI were significantly associated with a lower risk of vomiting, abdominal pain, bloody stools, and proteinuria in IgAV children.

## 1 Introduction

Immunoglobulin A vasculitis (IgAV), or Henoch–Schönlein’s purpura, is the most frequent systemic vasculitis in childhood with an incidence fluctuating from 3 to 27 cases per 100,000 children (1). Although the disease is most often self-limiting, various acute and chronic complications are possible. The skin, kidney, gastrointestinal tract, and joints are often involved, Gastrointestinal (GI) involvement is present in more than 50% children with IgAV. IgA nephritis (IgAVN) is reported in between 20 and 54% percent of children with IgAV and is more common in older children and adult (2, 3). However, the causes and risk factors remain unknown.

Research finds that underweight is an independent risk factor for kidney disease progression in IgA nephropathy (IgAN), which might be associated with malnutrition status and decreased complement 3 (C3) levels (4). In addition, the influence of obesity has been evaluated in various autoimmune and autoinflammatory diseases. Zhao et al. (5) showed that obesity was an independent risk factor for kidney involvement in IgAV. Obese children with IgAV seem to have higher erythrocyte sedimentation rate (ESR), hypertension, and severe kidney involvement; show delayed improvement of skin, joint, and kidney findings; these findings imply that increased adipose tissue contributes to the disease severity in IgAV probably via high levels of inflammatory mediators (6). A previous study has shown that patients with GI symptoms and IgAV have lower values of albumin and total serum proteins compared with patients without GI symptoms (7). The lower the values of these parameters, the higher the intensity of GI symptom. In a word, the above evidence emerged that nutrition status plays a non-substitutable role in the management of IgAV.

The IgAV is in the state of systemic immune inflammation characterized by involvements of skin, joint and gastrointestinal tract involvement, lots of immune indicators changing with ongoing immune inflammation process also have the potential to predict the development of IgAVN. In addition, it was reported that some common immune-inflammation index, such as neutrophil-to-lymphocyte ratio (NLR), monocyte-to-lymphocyte ratio (MLR), platelet-to-lymphocyte ratio (PLR) and systemic immune-inflammation index (SII), can also reflect the inflammation level and predict the occurrence of nephritis (8, 9), all of which are related to lymphocytes.

Meanwhile, a novel marker of nutrition and inflammation, the prognostic nutritional index (PNI), has been studied in various diseases. Originally used after GI surgery, PNI is now widely used to predict and evaluate GI disease. The PNI has also been used to predict other vasculitis diseases, such as Kawasaki disease (KD) and intravenous immunoglobulin nonresponse (10). Furthermore, some scholars have pointed out PNI is associated with renal function and pathologic lesions in IgAN patients (11). Since nutritional and inflammatory status is closely related to disease activity and adverse outcomes in immune-mediated diseases, measuring serum albumin levels and lymphocyte counts alone is susceptible to various confounding factors. The combination of these two parameters, the PNI, has shown significant clinical relevance in other vasculitis diseases such as KD and renal diseases. Therefore, we hypothesized that PNI may be significantly associated with the clinical manifestations of children with IgAV, and thus we investigated the relationship between PNI and common clinical features (including vomiting, abdominal pain, hematochezia, and proteinuria) in children with IgAV.

## 2 Methods

### 2.1 Study population

**Figure 1:**
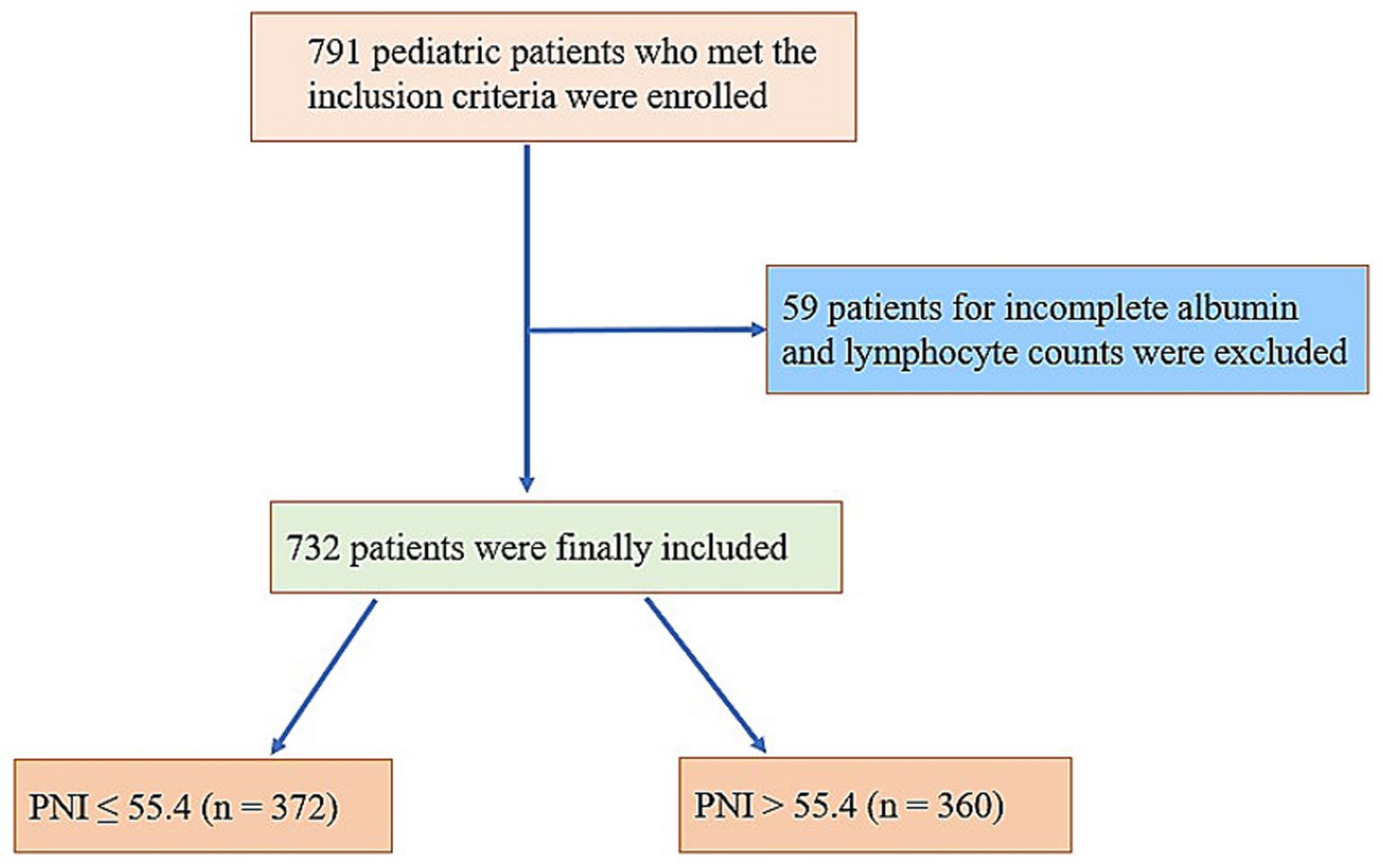
Flow chart of the study.

In this single-center retrospective cross-sectional study, we enrolled 732 pediatric patients (aged 1–15 years) diagnosed with IgAV at Xiangtan Central Hospital between January 2014 and December 2025 (Figure 1). All patients were consecutively enrolled inpatients, including those with first-episode and relapsed disease. Relapse referred to the reappearance of rash and abdominal pain after complete remission of symptoms for 1 month. Diagnosis of the disease was based on the EULAR/PRINTO/PRES criteria (12). Exclusion criteria: (1) Patients with severe liver or kidney failure were excluded; (2) Patients with malignant tumors were excluded; (3) Patients lacking baseline data including serum albumin and lymphocyte counts were excluded; (4) Patients without documented complications in clinical records were excluded. This study has been reviewed and approved by the Ethics Committee of Xiangtan Central Hospital Affiliated to Hunan University. Furthermore, the research protocol complied with the Declaration of Helsinki, and all patients provided informed consent.

### 2.2 Data collection

This study collected demographic data, clinical characteristics, and laboratory data from the electronic medical record system. Demographic data included age and sex. Clinical characteristics included time from onset to consultation, concurrent infection, rash severity, vomiting, abdominal pain, gastrointestinal bleeding, bloody stool, hematuria, proteinuria, and joint swelling/pain. Laboratory data include fibrinogen (FIB), activated partial thromboplastin time (APTT), anti-streptolysin O (ASO), serum Immunoglobulin G (IgG), Immunoglobulin A (IgA), and Immunoglobulin M (IgM), C3 and complement 4 (C4), uric acid (UA), serum creatinine (SCr), albumin, alanine aminotransferase (ALT), aspartate aminotransferase (AST), total bilirubin, C-reactive protein (CRP), ESR, white blood cell count (WBC), hemoglobin, and platelet count. All blood samples were collected before the initiation of glucocorticoid therapy.

### 2.3 Definition and grouping of PNI

The PNI was calculated using serum albumin concentration and lymphocyte count [PNI = albumin (g/L) + 5 × lymphocyte count (×10^9^/L)]. The PNI was analyzed not only as a raw continuous variable but also underwent standardization, where the standardized PNI = (raw PNI value – mean)/standard deviation (SD). In addition, analyses were performed based on the median, mean, tertile groupings, and quartile groupings of PNI.

### 2.4 Definition of complications

The analysis encompassed a range of clinical manifestations and complications associated with IgAV, including the presence of infections, severity of rashes, vomiting, abdominal pain, gastrointestinal bleeding, bloody stools, hematuria, proteinuria, and joint swelling and pain. Infection primarily pertains to respiratory tract infections, characterized by symptoms of upper respiratory tract infection upon initial hospital admission and confirmed by positive respiratory tract pathogen test results. Specifically, among the 146 infected children (19.9%), 44 had Group A Streptococcus infection, 24 were positive for Mycoplasma, 18 had Epstein–Barr virus infection, 12 had rhinovirus infection, 9 had influenza virus infection, 6 had adenovirus infection, and the remainder were mixed infections or sporadic cases of respiratory syncytial virus, pertussis, mumps, and other infections. Conventional symmetric hemorrhagic rashes localized to both lower limbs are classified as mild. Rashes that extend to the trunk, upper limbs, and wrists are categorized as moderate. In contrast, diffuse rashes that spread to the facial region, or those that rapidly progress to hemorrhagic bullae, ulcerative, or necrotic forms, are classified as severe (7). The medical history documented instances of vomiting, abdominal pain, and joint swelling/pain. Gastrointestinal bleeding is defined by the presence of hematemesis, melena, hematochezia, and positive fecal occult blood tests. Bloody stools refer specifically to hematochezia and positive fecal occult blood tests. Hematuria is defined as the presence of more than five red blood cells (RBC) in the field of vision, while proteinuria is indicated by levels exceeding 150 mg/24 h or a urinary albumin/creatinine ratio greater than 20 mg/mmol (or 0.2 mg/mg), or urine protein levels of at least 1^+^ (13).

### 2.5 Statistical analysis

This study employed SPSS 26.0 for statistical analysis. Prior to analysis, all continuous variables were assessed using the Shapiro–Wilk normality test. Normally distributed continuous variables were reported as mean ± SD, and independent samples t-tests were utilized for intergroup comparisons. Conversely, non-normally distributed continuous variables were expressed as median (interquartile range), with the Mann–Whitney *U* test applied for intergroup comparisons. A univariate analysis was conducted to evaluate the relationship between each variable and complications. Variables with *p* < 0.05 were subsequently selected to construct a multivariate analysis model for further assessment of the independent correlation between PNI and complications. For vomiting, Model 1 involved no covariates adjustment; Model 2 adjusted for WBC, SBP, ALT, UA, SCr, and APTT only; and Model 3 adjusted for WBC, SBP, ALT, UA, SCr, APTT, CRP, ESR, C4, IgA, and IgG. For abdominal pain, Model 1 involved no covariates adjustment; Model 2 adjusted for WBC, hemoglobin, platelet count, creatinine, and APTT only; and Model 3 adjusted for WBC, hemoglobin, platelet count, creatinine, APTT, ASO, ESR, complement C4, IgA, and IgG. For bloody stools, Model 1 involved no covariates adjustment; Model 2 adjusted for WBC, platelet count, systolic blood pressure, diastolic blood pressure, uric acid, and APTT only; and Model 3 adjusted for WBC, platelet count, systolic blood pressure, diastolic blood pressure, uric acid, APTT, CRP, ESR, IgG, and IgM. For proteinuria, Model 1 involved unadjusted covariates; Model 2 adjusted for uric acid, and APTT only; and Model 3 adjusted for uric acid, APTT, and IgG. In the fully adjusted model, a multicollinearity diagnosis was performed. Subsequently, multivariate subgroup analyses were performed based on age, sex, time from onset to medical consultation, and the presence of severe rash to further evaluate the stratified association between PNI and complications. Additionally, for missing values, we employed multiple imputation and complete-case analysis methods, using predictive mean matching for continuous variables and logistic regression for binary data. Two sensitivity analyses were also conducted to reassess the robustness of the association between PNI and vomiting, abdominal pain, bloody stools, and proteinuria. Finally, the correlation of PNI for vomiting, abdominal pain, bloody stools, and proteinuria was evaluated using ROC curves, with the area under the curve (AUC) and 95% confidence interval (95% CI) reported. All analyses in this study were two-sided tests, and a *p*-value < 0.05 was considered statistically significant.

## 3 Results

### 3.1 Clinical characteristics according to PNI median grouping

As shown in Table 1, a total of 732 patients were enrolled, including 372 (50.8%) in the low PNI group and 360 (49.2%) in the high PNI group. Compared with the low PNI group, the high PNI group had younger age, lower FIB, IgA, CRP, and ESR, as well as higher APTT, WBC, albumin, AST, and ALT, along with lower incidences of vomiting, abdominal pain, bloody stool, gastrointestinal bleeding, and proteinuria (*p* < 0.05).

**Table 1:** Clinical characteristics according to PNI median grouping.

Variables	Total population	Low PNI	High PNI	*p*-value
*N*	732	372	360	
Demographic characteristics				
Age (years)	7.21 ± 2.71	7.62 ± 2.61	6.79 ± 2.75	<0.001
Sex, *n* (%)				0.024
Male	401 (54.8%)	219 (54.6%)	182 (45.4%)	
Female	331 (45.2%)	153 (46.2%)	178 (53.8%)	
Clinical features				
Time from onset to consultation (days)	4.00 (2.00, 8.00)	4 0.00 (2.00, 7.00)	4.00 (2.00, 10.00)	0.222
Concurrent infections, *n* (%)	146 (19.9%)	82 (56.2%)	64 (43.8%)	0.149
Moderate to severe rash, *n* (%)	208 (28.4%)	111 (53.4%)	97 (46.6%)	0.385
Vomiting, *n* (%)	157 (21.4%)	105 (66.9%)	52 (33.1%)	<0.001
Abdominal pain, *n* (%)	385 (52.6%)	229 (59.5%)	156 (40.5%)	<0.001
Gastrointestinal bleeding, *n* (%)	130 (17.8%)	79 (60.8%)	51 (39.2%)	0.012
Bloody stool, *n* (%)	74 (10.1%)	49 (66.2%)	25 (33.8%)	0.005
Hematuria, *n* (%)	224 (30.6%)	106 (47.3%)	118 (52.7%)	0.209
Proteinuria, *n* (%)	66 (9.0%)	44 (66.7%)	22 (33.3%)	0.007
Joint swelling and pain, *n* (%)	510 (69.7%)	267 (52.4%)	243 (47.6%)	0.209
Laboratory findings				
FIB (g/L)	3.67 ± 0.99	3.79 ± 0.92	3.55 ± 1.06	0.001
APTT (s)	38.91 ± 6.32	38.44 ± 6.89	39.39 ± 5.64	0.043
ASO (U/mL)	110.50 (13.50, 271.48)	117.50 (13.50, 271.48)	101.50 (13.50, 271.48)	0.422
IgA (mg/mL)	2.40 (1.80, 2.79)	2.40 (1.84, 2.94)	2.38 (1.75, 2.60)	0.008
IgG (mg/mL)	11.32 ± 3.51	11.32 ± 4.03	11.31 ± 2.87	0.984
IgM (mg/mL)	1.23 (0.90, 1.35)	1.20 (0.87, 1.35)	1.25 (0.94, 1.37)	0.168
C4 (g/L)	0.26 (0.22, 0.30)	0.26 (0.22, 0.31)	0.26 (0.23, 0.29)	0.507
C3 (g/L)	1.22 ± 0.23	1.22 ± 0.25	1.23 ± 0.21	0.466
UA (μmol/L)	250.00 (208.00, 296.75)	252.00 (208.00, 296.75)	246.50 (207.25, 296.75)	0.797
SCr (μmol/L)	38.00 (31.00, 53.53)	38.00 (30.00, 53.00)	41.00 (31.00, 54.00)	0.100
ALB (g/L)	42.21 ± 3.93	40.61 ± 3.81	43.87 ± 3.31	<0.001
AST (U/L)	21.10 (17.70, 24.70)	20.10 (16.80, 23.58)	22.50 (18.60, 26.20)	<0.001
ALT (U/L)	8.70 (7.00, 11.68)	8.30 (6.60, 10.98)	9.50 (7.40, 12.28)	<0.001
TBIL (μmol/L)	5.10 (3.70, 6.90)	5.30 (3.73, 7.20)	4.95 (3.70, 6.60)	0.274
CRP (mg/L)	8.97 (3.08, 8.97)	8.97 (4.60, 9.01)	8.87 (1.97, 8.97)	<0.001
ESR (mm/h)	21.00 (12.00, 28.75)	22.00 (12.00, 31.00)	20.00 (11.00, 26.00)	0.016
WBC (×10^9^/L)	9.67 (7.78, 12.14)	9.28 (7.52, 11.73)	10.13 (8.10, 12.52)	0.003
HB (g/L)	124.44 ± 11.27	123.95 ± 11.85	124.96 ± 10.62	0.226
PLT (×10^9^/L)	335.00 (280.25, 401.00)	333.50 (274.00, 398.00)	339.00 (286.00, 405.00)	0.208

FIB, fibrinogen; APTT, activated partial thromboplastin time; ASO, anti-streptolysin O; C3, complement C3; C4, complement C4; UA, uric acid; SCr, serum creatinine; ALB, albumin; AST, aspartate aminotransferase; ALT, alanine aminotransferase; TBIL, total bilirubin; CRP, C-reactive protein; ESR, erythrocyte sedimentation rate; WBC, white blood cell; HB, hemoglobin; PLT, platelet count.

### 3.2 Univariate logistic regression analysis

As shown in Table 2, in the univariate Logistic regression analysis, WBC, hemoglobin, ALT, UA, Scr, APTT, CRP, ESR, C4, IgA, and IgG was significantly associated with the risk of vomiting (*p* < 0.05). WBC, hemoglobin, platelet count, Scr, APTT, ASO, ESR, C4, IgA, and IgG showed significant correlations with abdominal pain (*p* < 0.05). WBC, platelets, UA, APTT, CRP, ESR, IgG, and IgM demonstrated significant correlations with bloody stool (*p* < 0.05). UA, APTT, and IgG were also significantly correlated with proteinuria (*p* < 0.05).

**Table 2:** Univariate logistic regression analysis.

Variables	Vomiting		Abdominal pain		Bloody stool		Proteinuria	
	OR (95% CI)	*P*-value	OR (95% CI)	*P*-value	OR (95% CI)	*P*-value	OR (95% CI)	*P*-value
Age	0.978 (0.915, 1.044)	0.505	1.005 (0.953, 1.061)	0.848	0.976 (0.892, 1.068)	0.600	1.108 (1.013, 1.213)	0.025
Male	0.849 (0.596, 1.210)	0.365	0.980 (0.732, 1.312)	0.893	1.402 (0.855, 2.298)	0.180	0.866 (0.522, 1.436)	0.576
Time from onset to consultation	1.002 (0.998, 1.005)	0.393	1.003 (0.998, 1.008)	0.281	0.996 (0.982, 1.011)	0.611	1.003 (0.999, 1.007)	0.106
Concurrent infections	0.796 (0.502, 1.262)	0.332	0.849 (0.590, 1.220)	0.375	0.930 (0.504, 1.715)	0.816	0.983 (0.521, 1.856)	0.958
FIB	1.059 (0.894, 1.255)	0.505	0.967 (0.835, 1.119)	0.651	0.858 (0.650, 1.132)	0.278	1.190 (0.966, 1.464)	0.101
APTT	0.908 (0.875, 0.941)	<0.001	0.949 (0.925. 0.974)	<0.001	0.909 (0.865, 0.954)	<0.001	0.944 (0.900, 0.991)	0.020
ASO	1.000 (0.999, 1.000)	0.260	0.999 (0.999, 1.000)	<0.001	1.000 (0.999, 1.000)	0.604	1.000 (0.999, 1.000)	0.625
IgA	0.786 (0.638, 0.969)	0.024	0.796 (0.679, 0.933)	0.005	0.886 (0.674, 1.163)	0.383	1.077 (0.772, 1.314)	0.958
IgG	0.885 (0.830, 0.943)	<0.001	0.884 (0.842, 0.928)	<0.001	0.893 (0.819, 0.975)	0.011	0.870 (0.791, 0.957)	0.004
IgM	0.741 (0.492, 1.118)	0.153	1.017 (0.940, 1.101)	0.668	0.390 (0.204, 0.747)	0.005	1.081 (0.972, 1.204)	0.151
C4	5.890 (1.027, 33.778)	0.047	8.668 (1.634, 45.987)	0.011	0.835 (0.070, 9.891)	0.886	0.325 (0.019, 5.577)	0.439
C3	0.964 (0.454, 2.049)	0.924	1.249 (0.671, 2.323)	0.485	0.852 (0.304, 2.384)	0.760	0.827 (0.280, 2.443)	0.731
UA	1.003 (1.001, 1.005)	0.003	1.002 (1.000, 1.003)	0.121	1.004 (1.002, 1.007)	0.001	1.004 (1.001, 1.007)	0.009
SCr	0.978 (0.964, 0.992)	0.003	0.986 (0.974, 0.997)	0.015	0.982 (0.963, 1.002)	0.078	1.005 (0.985, 1.025)	0.654
ALB	0.918 (0.878, 0.960)	<0.001	0.963 (0.927, 0.979)	0.046	0.922 (0.870, 0.977)	0.006	0.947 (0.890, 1.008)	0.085
AST	0.979 (0.955, 1.004)	0.094	0.995 (0.979, 1.011)	0.514	0.962 (0.925, 1.001)	0.058	0.975 (0.938, 1.013)	0.189
ALT	0.954 (0.920, 0.989)	0.011	0.996 (0.979, 1.012)	0.617	0.971 (0.929, 1.015)	0.192	0.998 (0.968, 1.028)	0.872
TBIL	1.032 (0.974, 1.093)	0.284	1.043 (0.992, 1.098)	0.100	1.042 (0.966, 1.123)	0.287	1.047 (0.968, 1.132)	0.253
CRP	1.016 (1.002, 1.031)	0.028	1.007 (0.993, 1.021)	0.335	1.025 (1.009, 1.041)	0.002	1.012 (0.993, 1.031)	0.211
ESR	0.988 (0.976, 0.999)	0.033	0.985 (0.977, 0.994)	<0.001	0.976 (0.957, 0.994)	0.010	0.995 (0.980, 1.010)	0.508
WBC	1.139 (1.091, 1.189)	<0.001	1.119 (1.073, 1.168)	<0.001	1.144 (1.088, 1.204)	<0.001	0.991 (0.930, 1.056)	0.776
HB	1.027 (1.011, 1.044)	0.001	1.026 (1.013, 1.040)	<0.001	1.020 (0.998, 1.043)	0.072	1.018 (0.995, 1.041)	0.134
PLT	1.002 (1.000, 1.003)	0.064	1.002 (1.000, 1.003)	0.035	1.004 (1.002, 1.006)	<0.001	1.000 (0.998, 1.002)	0.974

FIB, fibrinogen; APTT, activated partial thromboplastin time; ASO, anti-streptolysin O; IgA, immunoglobulin A; IgG, immunoglobulin G; IgM, immunoglobulin M; C3, complement C3; C4, complement C4; UA, uric acid; SCr, serum creatinine; ALB, albumin; AST, aspartate aminotransferase; ALT, alanine aminotransferase; TBIL, total bilirubin; CRP, C-reactive protein; ESR, erythrocyte sedimentation rate; WBC, white blood cell; HB, hemoglobin; PLT, platelet count.

### 3.3 Multivariate logistic regression analysis

As demonstrated in Table 3, in the unadjusted Model 1, the partially adjusted Model 2, and the fully adjusted Model 3, the PNI was significantly associated with a reduced risk of vomiting, abdominal pain, bloody stools, and proteinuria. And in the fully adjusted model, the variance inflation factor for PNI and other inflammatory and immune markers were all lower than 2. Specifically, in the fully adjusted Model 3, each unit increase and one SD increase in PNI corresponded to a 9.4% (OR = 0.906, 95% CI: 0.877–0.937) and a 50.5% (OR = 0.495, 95% CI: 0.391–0.627) reduction in the risk of vomiting, respectively. Moreover, when comparing the low PNI group to the high PNI group, the latter exhibited a 55.4% (OR = 0.446, 95% CI: 0.298–0.667) reduction in vomiting risk.

**Table 3:** Multivariate logistic regression analysis.

Variables	Model 1		Model 2		Model 3	
	OR (95% CI)	*P*-value	OR (95% CI)	*P*-value	OR (95% CI)	*P*-value
Vomiting						
PNI	0.919 (0.891, 0.947)	<0.001	0.913 (0.884, 0.943)	<0.001	0.906 (0.877, 0.937)	<0.001
Standardized PNI	0.547 (0.441, 0.678)	<0.001	0.522 (0.416, 0.656)	<0.001	0.495 (0.391, 0.627)	<0.001
PNI (High vs. Low)	0.429 (0.296, 0.622)	<0.001	0.440 (0.295, 0.655)	<0.001	0.446 (0.298, 0.667)	<0.001
Abdominal pain						
PNI	0.957 (0.936, 0.978)	<0.001	0.943 (0.921, 0.965)	<0.001	0.933 (0.910, 0.957)	<0.001
Standardized PNI	0.730 (0.626, 0.851)	<0.001	0.656 (0.555, 0.776)	<0.001	0.611 (0.512, 0.730)	<0.001
PNI (High vs. Low)	0.478 (0.355, 0.641)	<0.001	0.430 (0.314, 0.586)	<0.001	0.396 (0.286, 0.548)	<0.001
Bloody stool						
PNI	0.952 (0.917, 0.989)	0.011	0.956 (0.920, 0.993)	0.022	0.951 (0.913, 0.990)	0.014
Standardized PNI	0.705 (0.538, 0.924)	0.011	0.725 (0.551, 0.954)	0.022	0.697 (0.522, 0.930)	0.014
PNI (High vs. Low)	0.492 (0.297, 0.815)	0.006	0.511 (0.299, 0.873)	0.014	0.478 (0.276, 0.830)	0.009
Proteinuria						
PNI	0.933 (0.895, 0.972)	<0.001	0.930 (0.891, 0.971)	<0.001	0.934 (0.895, 0.975)	0.002
Standardized PNI	0.608 (0.453, 0.818)	<0.001	0.596 (0.439, 0.809)	<0.001	0.615 (0.453, 0.837)	0.002
PNI (High vs. Low)	0.485 (0.284, 0.828)	0.008	0.495 (0.289, 0.848)	0.010	0.530 (0.308, 0.915)	0.023

For vomiting, Model 1 involved no covariates adjustment; Model 2 adjusted for WBC, SBP, ALT, UA, SCr, and APTT only; and Model 3 adjusted for WBC, SBP, ALT, UA, SCr, APTT, CRP, ESR, C4, IgA, and IgG.

For abdominal pain, Model 1 involved no covariates adjustment; Model 2 adjusted for WBC, HB, PLT, SCr, and APTT only; and Model 3 adjusted for WBC, HB, PLT, SCr, APTT, ASO, ESR, C4, IgA, and IgG.

For bloody stools, Model 1 involved no covariates adjustment; Model 2 adjusted for WBC, PLT, SBP, DBP, UA, and APTT only; and Model 3 adjusted for WBC, PLT, SBP, DBP, UA, APTT, CRP, ESR, IgG, and IgM.

For proteinuria, Model 1 involved unadjusted covariates; Model 2 adjusted for UA, urea nitrogen, and APTT only; and Model 3 adjusted for UA, urea nitrogen, APTT, and IgG.

WBC, white blood cell; SBP, systolic blood pressure; ALT, alanine aminotransferase; UA, uric acid; SCr, serum creatinine; APTT, activated partial thromboplastin time; CRP, C-reactive protein; ESR, erythrocyte sedimentation rate; C4, complement C4; IgA, immunoglobulin A; IgG, immunoglobulin G; IgM, immunoglobulin M; PLT, platelet count; DBP, diastolic blood pressure.

Similarly, for each unit and SD increase in PNI, the risk of abdominal pain decreased by 6.7% (OR = 0.933, 95% CI: 0.910–0.957) and 38.9% (OR = 0.611, 95% CI: 0.512–0.730), respectively. Additionally, the high PNI group showed a 60.4% reduction in abdominal pain risk compared to the low PNI group (OR = 0.396, 95% CI: 0.286–0.548).

Furthermore, for each unit and SD increase in PNI, the risk of bloody stools decreased by 4.9% (OR = 0.951, 95% CI: 0.913–0.990) and 30.3% (OR = 0.697, 95% CI: 0.522–0.930), respectively. In comparison to the low PNI group, the high PNI group had a 52.2% lower risk of bloody stools (OR = 0.478, 95% CI: 0.276–0.830). Lastly, for each unit and SD increase in PNI, the risk of proteinuria decreased by 6.6% (OR = 0.934, 95% CI: 0.895–0.975) and 38.5% (OR = 0.615, 95% CI: 0.453–0.837), respectively. Furthermore, the high PNI group demonstrated a 47.0% reduction in proteinuria risk compared to the low PNI group (OR = 0.530, 95% CI: 0.308–0.915).

### 3.4 Subgroup analysis

As demonstrated in Table 4, for vomiting, in the subgroup aged ≤ 7 years, each unit increase and one SD increase in PNI were associated with an 8.1% (OR = 0.919) and 45.2% (OR = 0.548) reduction in vomiting risk, respectively. Compared with the low PNI group, the high PNI group showed a 52.0% (OR = 0.480) reduction in vomiting risk. In the subgroup aged > 7 years, each unit increase and one SD increase in PNI were associated with an 11.8% (OR = 0.882) and 59.3% (OR = 0.407) reduction in vomiting risk, respectively. Compared with the low PNI group, the high PNI group had a 72.4% lower risk of vomiting (OR = 0.276).

**Table 4:** Subgroup analysis.

Variables	PNI		Standardized PNI		PNI (High vs. Low)	
	OR (95% CI)	*P*-value	OR (95% CI)	*P*-value	OR (95% CI)	*P*-value
Vomiting						
Age						
≤7 years	0.919 (0.884, 0.956)	<0.001	0.548 (0.414, 0.715)	<0.001	0.480 (0.289, 0.798)	0.005
>7 years	0.882 (0.833, 0.933)	<0.001	0.407 (0.272, 0.610)	<0.001	0.276 (0.134, 0.566)	<0.001
*P* for interaction		0.242		0.230		0.219
Sex						
Male	0.923 (0.884, 0.963)	<0.001	0.563 (0.414, 0.765)	<0.001	0.495 (0.287, 0.853)	0.011
Female	0.891 (0.848, 0.937)	<0.001	0.441 (0.309, 0.629)	<0.001	0.342 (0.187, 0.626)	<0.001
*P* for interaction		0.294		0.308		0.374
Time from onset to consultation						
≤4 days	0.908 (0.864, 0.953)	<0.001	0.501 (0.354, 0.710)	<0.001	0.487 (0.264, 0.900)	0.022
>4 days	0.921 (0.881, 0.963)	<0.001	0.556 (0.405, 0.762)	<0.001	0.396 (0.229, 0.686)	<0.001
*P* for interaction		0.674		0.667		0.624
Moderate to severe rash						
Yes	0.896 (0.847, 0.949)	<0.001	0.459 (0.306, 0.688)	<0.001	0.293 (0.140, 0.610)	0.001
No	0.914 (0.878, 0.950)	<0.001	0.525 (0.397, 0.694)	<0.001	0.473 (0.292, 0.765)	0.002
P for interaction		0.575		0.589		0.285
Abdominal pain						
Age						
≤7 years	0.936 (0.908, 0.964)	<0.001	0.623 (0.503, 0.722)	<0.001	0.377 (0.247, 0.574)	<0.001
>7 years	0.919 (0.877, 0.964)	<0.001	0.548 (0.391, 0.769)	<0.001	0.416 (0.244, 0.711)	0.001
*P* for interaction		0.516		0.516		0.772
Sex						
Male	0.951 (0.919, 0.985)	0.004	0.701 (0.549, 0.895)	0.004	0.436 (0.275, 0.689)	<0.001
Female	0.902 (0.868, 0.937)	<0.001	0.479 (0.364, 0.629)	<0.001	0.303 (0.187, 0.491)	<0.001
*P* for interaction		0.044		0.041		0.285
Time from onset to consultation						
≤4 days	0.935 (0.903, 0.968)	<0.001	0.618 (0.483, 0.790)	<0.001	0.352 (0.220, 0.562)	<0.001
>4 days	0.924 (0.889, 0.959)	<0.001	0.567 (0.433, 0.744)	<0.001	0.395 (0.246, 0.635)	<0.001
*P* for interaction		0.653		0.648		0.734
Moderate to severe rash						
Yes	0.944 (0.902, 0.989)	0.014	0.664 (0.479, 0.922)	0.014	0.393 (0.209, 0.741)	0.004
No	0.928 (0.901, 0.956)	<0.001	0.586 (0.475, 0.723)	<0.001	0.410 (0.281, 0.599)	<0.001
*P* for interaction		0.542		0.529		0.912
Bloody stool						
Age						
≤7 years	0.934 (0.889, 0.982)	0.008	0.616 (0.431, 0.881)	0.008	0.396 (0.199, 0.790)	0.009
>7 years	0.992 (0.921, 1.068)	0.827	0.943 (0.557, 1.597)	0.827	0.772 (0.294, 2.028)	0.600
*P* for interaction		0.184		0.190		0.271
Sex						
Male	0.976 (0.924, 1.030)	0.374	0.838 (0.568, 1.236)	0.374	0.482 (0.227, 1.023)	0.057
Female	0.920 (0.854, 0.991)	0.029	0.553 (0.326, 0.940)	0.029	0.597 (0.245, 1.455)	0.257
*P* for interaction		0.208		0.215		0.719
Time from onset to consultation						
≤4 days	0.945 (0.885, 1.008)	0.086	0.666 (0.419, 1.060)	0.086	0.448 (0.176, 1.140)	0.092
>4 days	0.981 (0.928, 1.038)	0.505	0.873 (0.597, 1.300)	0.505	0.610 (0.292, 1.273)	0.188
*P* for interaction		0.395		0.384		0.610
Moderate to severe rash						
Yes	0.993 (0.939, 1.050)	0.807	0.952 (0.640, 1.416)	0.807	0.875 (0.380, 2.018)	0.755
No	0.923 (0.873, 0.997)	0.006	0.566 (0.378, 0.848)	0.006	0.319 (0.138, 0.736)	0.007
*P* for interaction		0.099		0.072		0.095
Proteinuria						
Age						
≤7 years	0.961 (0.907, 1.019)	0.182	0.754 (0.497, 1.142)	0.182	0.580 (0.260, 1.296)	0.184
>7 years	0.916 (0.855, 0.981)	0.013	0.536 (0.328, 0.875)	0.013	0.532 (0.243, 1.167)	0.116
*P* for interaction		0.298		0.298		0.881
Sex						
Male	0.967 (0.911, 1.026)	0.263	0.785 (0.514, 1.199)	0.263	0.598 (0.278, 1.285)	0.188
Female	0.899 (0.842, 0.960)	0.001	0.475 (0.297, 0.760)	0.002	0.489 (0.222, 1.077)	0.076
*P* for interaction		0.107		0.119		0.719
Time from onset to consultation						
≤4 days	0.929 (0.874, 0.987)	0.018	0.590 (0.382, 0.913)	0.018	0.433 (0.190, 0.984)	0.046
>4 days	0.945 (0.890, 1.003)	0.063	0.667 (0.435, 1.022)	0.063	0.630 (0.302, 1.317)	0.200
*P* for interaction		0.697		0.689		0.503
Moderate to severe rash						
Yes	0.956 (0.889, 1.027)	0.220	0.725 (0.434, 1.212)	0.220	0.693 (0.271, 1.768)	0.442
No	0.926 (0.877, 0.979)	0.006	0.580 (0.392, 0.857)	0.006	0.503 (0.254, 0.994)	0.048
*P* for interaction		0.490		0.497		0.589

For vomiting, subgroup analyses were adjusted for WBC, SBP, ALT, UA, SCr, APTT, CRP, ESR, C4, IgA, and IgG.

For abdominal pain, subgroup analyses were adjusted for WBC, HB, PLT, SCr, APTT, ASO, ESR, C4, IgA, and IgG.

For bloody stool, subgroup analyses were adjusted for WBC, PLT, SBP, DBP, UA, APTT, CRP, ESR, IgG, and IgM.

For proteinuria, subgroup analyses were adjusted for UA, urea nitrogen, APTT, and IgG.

WBC, white blood cell; SBP, systolic blood pressure; ALT, alanine aminotransferase; UA, uric acid; SCr, serum creatinine; APTT, activated partial thromboplastin time; CRP, C-reactive protein; ESR, erythrocyte sedimentation rate; C4, complement C4; IgA, immunoglobulin A; IgG, immunoglobulin G; IgM, immunoglobulin M; PLT, platelet count; DBP, diastolic blood pressure.

Among males, each unit increase and each SD increase in PNI were associated with a 7.7% (OR = 0.923) and 43.7% (OR = 0.563) reduction in vomiting risk, respectively. Compared with the low PNI group, the high PNI group showed a 50.5% reduction in vomiting risk (OR = 0.495). In females, each unit increase and one SD increase in PNI were associated with a 10.9% (OR = 0.891) and 55.9% (OR = 0.441) reduction in vomiting risk, respectively. Moreover, compared with the low PNI group, the high PNI group demonstrated a 65.8% decrease in vomiting risk (OR = 0.342).

In patients with a time from onset to consultation ≤ 4 days, each unit increase and one SD increase in PNI were associated with a 9.2% (OR = 0.908) and 49.9% (OR = 0.501) reduction in vomiting risk, respectively. Compared with the low PNI group, the high PNI group showed a 51.3% (OR = 0.487) decrease in vomiting risk. In patients with a time from onset to consultation > 4 days, each unit increase and one SD increase in PNI were associated with a 7.9% (OR = 0.921) and 44.4% (OR = 0.556) reduction in vomiting risk, respectively. Compared with the low PNI group, the high PNI group showed a 60.4% reduction in vomiting risk (OR = 0.396).

In moderate-to-severe rash, each unit increase and one SD increase in PNI were associated with a 10.4% (OR = 0.896) and 54.1% (OR = 0.459) reduction in vomiting risk, respectively. Moreover, compared with the low PNI group, the high PNI group demonstrated a 70.7% decrease in vomiting risk (OR = 0.293). In cases without moderate to severe rash, each unit increase and one SD increase in PNI were associated with an 8.6% (OR = 0.914) and 47.5% (OR = 0.525) reduction in vomiting risk, respectively. Moreover, compared to the low PNI group, the high PNI group showed a 52.7% (OR = 0.473) decrease in vomiting risk.

For abdominal pain, in the subgroup aged ≤ 7 years, each unit increase and one SD increase in PNI were associated with a 6.4% (OR = 0.936) and 37.7% (OR = 0.623) reduction in abdominal pain risk, respectively. Compared with the low PNI group, the high PNI group showed a 62.3% (OR = 0.377) reduction in abdominal pain risk. In the subgroup aged > 7 years, each unit increase and one SD increase in PNI were associated with an 8.1% (OR = 0.919) and 45.2% (OR = 0.548) reduction in abdominal pain risk, respectively. Compared with the low PNI group, the high PNI group had a 58.4% reduction in the risk of abdominal pain (OR = 0.416).

In males, each unit increase and one SD increase in PNI were associated with a 4.9% (OR = 0.951) and 29.9% (OR = 0.701) reduction in the risk of abdominal pain, respectively. Compared with the low PNI group, the high PNI group showed a 56.4% reduction in abdominal pain risk (OR = 0.436). Among females, each unit increase and one SD increase in PNI were associated with a 9.8% (OR = 0.902) and 52.1% (OR = 0.479) reduction in abdominal pain risk, respectively. Additionally, compared with the low PNI group, the high PNI group demonstrated a 69.7% reduction in abdominal pain risk (OR = 0.303).

In patients with a time from onset to consultation ≤ 4 days, each unit increase and each SD increase in PNI were associated with a 6.5% (OR = 0.935) and 38.2% (OR = 0.618) reduction in abdominal pain risk, respectively. Moreover, compared to the low PNI group, the high PNI group showed a 64.8% (OR = 0.352) reduction in abdominal pain risk. In patients with a time from onset to consultation > 4 days, each unit increase and each SD increase in PNI were associated with a 7.6% (OR = 0.924) and 43.3% (OR = 0.567) reduction in abdominal pain risk, respectively. Compared with the low PNI group, the high PNI group showed a 60.5% reduction in abdominal pain risk (OR = 0.395).

In moderate-to-severe rash, each unit increase and one SD increase in PNI were associated with a 5.6% (OR = 0.944) and 33.6% (OR = 0.664) reduction in abdominal pain risk, respectively. Additionally, compared with the low PNI group, the high PNI group demonstrated a 60.7% reduction in abdominal pain risk (OR = 0.393). In the absence of moderate-to-severe rash, each unit increase and one SD increase in PNI were associated with a 7.2% (OR = 0.9928) and 41.4% (OR = 0.586) reduction in abdominal pain risk, respectively. Moreover, compared to the low PNI group, the high PNI group exhibited a 59.0% (OR = 0.410) lower risk of abdominal pain.

For bloody stools, in the subgroup aged ≤ 7 years, each unit increase and one SD increase in PNI were associated with a 6.6% (OR = 0.934) and 38.4% (OR = 0.616) reduction in the risk of bloody stools, respectively. Moreover, compared to the low PNI group, the high PNI group showed a 60.4% (OR = 0.396) reduction in the risk of bloody stools. In females, each unit increase and one SD increase in PNI were associated with an 8.0% (OR = 0.920) and 44.7% (OR = 0.553) reduction in bloody stool risk, respectively. In non-moderate-to-severe rash cases, each unit increase and one SD increase in PNI corresponded to a 7.7% (OR = 0.923) and 43.4% (OR = 0.566) decrease in bloody stool risk, respectively. Moreover, compared to the low PNI group, the high PNI group showed a 68.1% (OR = 0.319) reduction in bloody stool risk.

For proteinuria, in the subgroup aged over 7 years, each unit increase and one SD increase in PNI were associated with an 8.4% reduction (OR = 0.916) and a 46.4% reduction (OR = 0.536) in proteinuria risk, respectively. Among females, each unit increase and one SD increase in PNI were associated with a 10.1% reduction (OR = 0.899) and a 52.5% reduction (OR = 0.475) in proteinuria risk, respectively. In patients with a time from onset to consultation ≤ 4 days, each unit increase and one SD increase in PNI were associated with a 7.1% (OR = 0.929) and 41.0% (OR = 0.590) reduction in proteinuria risk, respectively. Compared with the low PNI group, the high PNI group showed a 56.7% (OR = 0.433) lower risk of proteinuria. In patients without moderate to severe rash, each unit increase and one SD increase in PNI were associated with a 7.4% (OR = 0.926) and 42.0% (OR = 0.580) reduction in proteinuria risk, respectively. Compared with the low PNI group, the high PNI group showed a 49.7% reduction in proteinuria risk (OR = 0.503).

Furthermore, in subgroup analyses, interaction tests were performed. PNI showed a negative correlation with the four clinical symptoms, and no significant interaction *p*-values were observed. No significant differences in the PNI effect were found across subgroups, indicating that the negative correlation was similar among different populations.

### 3.5 Sensitivity analysis: exclude patients with concurrent infections

As shown in Table 5, in the multivariate logistic regression analysis after excluding patients with co-infections, each unit increase and each SD increase in PNI were associated with a 10.5% (OR = 0.895, 95% CI: 0.861–0.930) and 54.7% (OR = 0.453, 95% CI: 0.345–0.594) reduction in vomiting risk, respectively. Moreover, compared with the low PNI group, the high PNI group showed a 60.1% (OR = 0.399, 95% CI: 0.253–0.628) decreased risk of vomiting.

**Table 5:** Sensitivity analysis: exclude patients with concurrent infections.

Variables	Model 1		Model 2		Model 3	
	OR (95% CI)	*P*-value	OR (95% CI)	*P*-value	OR (95% CI)	*P*-value
Vomiting						
PNI	0.912 (0.882, 0.944)	<0.001	0.907 (0.875, 0.940)	<0.001	0.895 (0.861, 0.930)	<0.001
Standardized PNI	0.520 (0.408, 0.622)	<0.001	0.497 (0.385, 0.643)	<0.001	0.453 (0.345, 0.594)	<0.001
PNI (High vs. Low)	0.397 (0.263, 0.597)	<0.001	0.423 (0.272, 0.656)	<0.001	0.399 (0.253, 0.628)	<0.001
Abdominal pain						
PNI	0.961 (0.939, 0.984)	<0.001	0.948 (0.924, 0.973)	<0.001	0.933 (0.907, 0.959)	<0.001
Standardized PNI	0.753 (0.637, 0.890)	<0.001	0.685 (0.569, 0.823)	<0.001	0.609 (0.499, 0.742)	<0.001
PNI (High vs. Low)	0.485 (0.348, 0.674)	<0.001	0.450 (0.316, 0.639)	<0.001	0.392 (0.270, 0.568)	<0.001
Bloody stool						
PNI	0.946 (0.907, 0.987)	0.011	0.950 (0.910, 0.991)	0.018	0.940 (0.898, 0.983)	0.007
Standardized PNI	0.675 (0.499, 0.914)	0.011	0.693 (0.512, 0.939)	0.018	0.642 (0.466, 0.886)	0.007
PNI (High vs. Low)	0.491 (0.281, 0.858)	0.012	0.510 (0.285, 0.913)	0.024	0.491 (0.268, 0.900)	0.021
Proteinuria						
PNI	0.926 (0.884, 0.971)	0.001	0.929 (0.884, 0.975)	0.003	0.931 (0.886, 0.978)	0.004
Standardized PNI	0.579 (0.415, 0.808)	0.001	0.590 (0.416, 0.837)	0.003	0.599 (0.421, 0.851)	0.004
PNI (High vs. Low)	0.430 (0.236, 0.748)	0.006	0.476 (0.257, 0.882)	0.018	0.499 (0.268, 0.928)	0.028

For vomiting, Model 1 involved no covariates adjustment; Model 2 adjusted for WBC, SBP, ALT, UA, SCr, and APTT only; and Model 3 adjusted for WBC, SBP, ALT, UA, SCr, APTT, CRP, ESR, C4, IgA, and IgG.

For abdominal pain, Model 1 involved no covariates adjustment; Model 2 adjusted for WBC, HB, PLT, SCr, and APTT only; and Model 3 adjusted for WBC, HB, PLT, SCr, APTT, ASO, ESR, C4, IgA, and IgG.

For bloody stools, Model 1 involved no covariates adjustment; Model 2 adjusted for WBC, PLT, SBP, DBP, UA, and APTT only; and Model 3 adjusted for WBC, PLT, SBP, DBP, UA, APTT, CRP, ESR, IgG, and IgM.

For proteinuria, Model 1 involved unadjusted covariates; Model 2 adjusted for UA, urea nitrogen, and APTT only; and Model 3 adjusted for UA, urea nitrogen, APTT, and IgG.

WBC, white blood cell; SBP, systolic blood pressure; ALT, alanine aminotransferase; UA, uric acid; SCr, serum creatinine; APTT, activated partial thromboplastin time; CRP, C-reactive protein; ESR, erythrocyte sedimentation rate; C4, complement C4; IgA, immunoglobulin A; IgG, immunoglobulin G; IgM, immunoglobulin M; PLT, platelet count; DBP, diastolic blood pressure.

For abdominal pain, each unit increase and one SD increase in PNI were associated with a 6.7% (OR = 0.933, 95% CI: 0.907–0.959) and 39.1% (OR = 0.609, 95% CI: 0.499–0.742) reduction in abdominal pain risk, respectively. Moreover, compared with the low PNI group, the high PNI group showed a 60.8% (OR = 0.392, 95% CI: 0.270–0.568) reduction in abdominal pain risk.

For bloody stools, each unit increase and one SD increase in PNI were associated with a 6.0% (OR = 0.940, 95% CI: 0.898–0.983) and 35.8% (OR = 0.642, 95% CI: 0.466–0.886) reduction in bloody stool risk, respectively. Compared with the low PNI group, the high PNI group showed a 50.9% reduction in bloody stool risk (OR = 0.491, 95% CI: 0.268–0.900).

For proteinuria, each unit increase and one SD increase in PNI were associated with a 6.9% (OR = 0.931, 95% CI: 0.886–0.978) and 40.1% (OR = 0.599, 95% CI: 0.421–0.851) reduction in proteinuria risk, respectively. Moreover, compared with the low PNI group, the high PNI group exhibited a 50.1% reduction in proteinuria risk (OR = 0.499, 95% CI: 0.268–0.928).

### 3.6 Sensitivity analysis: group by mean, tertiles, and quartiles of PNI

We further evaluated the consistency of the associations among different PNI grouping methods (mean, tertiles, and quartiles). As shown in Table 6, In the fully adjusted Model 3, when grouped by the mean value, compared with the low PNI group, the risk of vomiting in the high PNI group was reduced by 57.9% (OR = 0.421). When grouped by PNI tertiles, the risk of vomiting in the T3 group was reduced by 64.9% (OR = 0.351) compared to the T1 group. When grouped by PNI quartiles, the risk in the Q4 group was reduced by 77.9% (OR = 0.221) compared to the Q1 group. For abdominal pain, when grouped by the mean value, compared with the low PNI group, the risk of abdominal pain in the high PNI group was reduced by 59.2% (OR = 0.408). The risk of abdominal pain in the T3 group was reduced by 63.1% (OR = 0.369) compared to the T1 group, respectively. When grouped by PNI quartiles, the risk in the Q4 group was reduced by 73.3% (OR = 0.267) compared to the Q1 group. For bloody stool, when grouped by the mean value, compared with the low PNI group, the risk of bloody stool in the high PNI group was reduced by 49.4% (OR = 0.506). When grouped by PNI tertiles and quartiles, Model 3 was not statistically significant but showed the same protective trend for bloody stools (see details in Supplementary Table 6). For proteinuria, when grouped by the mean value, compared with the low PNI group, the risk of proteinuria in the high PNI group was reduced by 51.5% (OR = 0.485). When grouped by PNI tertiles, the risk of proteinuria in the T3 group was reduced by 59.6% (OR = 0.404) compared to the T1 group. When grouped by PNI quartiles, the risk of proteinuria in the Q4 group was reduced by 73.4% (OR = 0.266) compared to the Q1 group. In short, regardless of the classification scheme used, a higher PNI level consistently exerts a protective effect against vomiting, abdominal pain, bloody stools, and proteinuria. This effect does not change with different PNI classification criteria, emphasizing the consistency of results across different methods.

**Table 6:** Sensitivity analysis: group by mean, tertiles, and quartiles of PNI.

Variables	Model 3	
	OR (95% CI)	*P*-value
Vomiting		
Mean	0.421 (0.280, 0.634)	<0.001
Tertiles (T3 vs. T1)	0.351 (0.215, 0.575)	<0.001
Quartiles (Q4 vs. Q1)	0.221 (0.120, 0.405)	<0.001
Abdominal pain		
Mean	0.408 (0.295, 0.565)	<0.001
Tertiles (T3 vs. T1)	0.369 (0.246, 0.551)	<0.001
Quartiles (Q4 vs. Q1)	0.267 (0.166, 0.430)	<0.001
Bloody stool		
Mean	0.506 (0.292, 0.877)	0.015
Tertiles (T3 vs. T1)	0.531 (0.276, 1.022)	0.058
Quartiles (Q4 vs. Q1)	0.474 (0.220, 1.020)	0.056
Proteinuria		
Mean	0.485 (0.278, 0.847)	0.011
Tertiles (T3 vs. T1)	0.404 (0.199, 0.819)	0.012
Quartiles (Q4 vs. Q1)	0.266 (0.111, 0.638)	0.003

For vomiting, Model 3 adjusted for WBC, SBP, ALT, UA, SCr, APTT, CRP, ESR, C4, IgA, and IgG.

For abdominal pain, Model 3 adjusted for WBC, HB, PLT, SCr, APTT, ASO, ESR, C4, IgA, and IgG.

For bloody stools, Model 3 adjusted for WBC, PLT, SBP, DBP, UA, APTT, CRP, ESR, IgG, and IgM.

For proteinuria, Model 3 adjusted for UA, urea nitrogen, APTT, and IgG.

WBC, white blood cell; SBP, systolic blood pressure; ALT, alanine aminotransferase; UA, uric acid; SCr, serum creatinine; APTT, activated partial thromboplastin time; CRP, C-reactive protein; ESR, erythrocyte sedimentation rate; C4, complement C4; IgA, immunoglobulin A; IgG, immunoglobulin G; IgM, immunoglobulin M; PLT, platelet count; DBP, diastolic blood pressure.

### 3.7 ROC analysis

**Figure 2:**
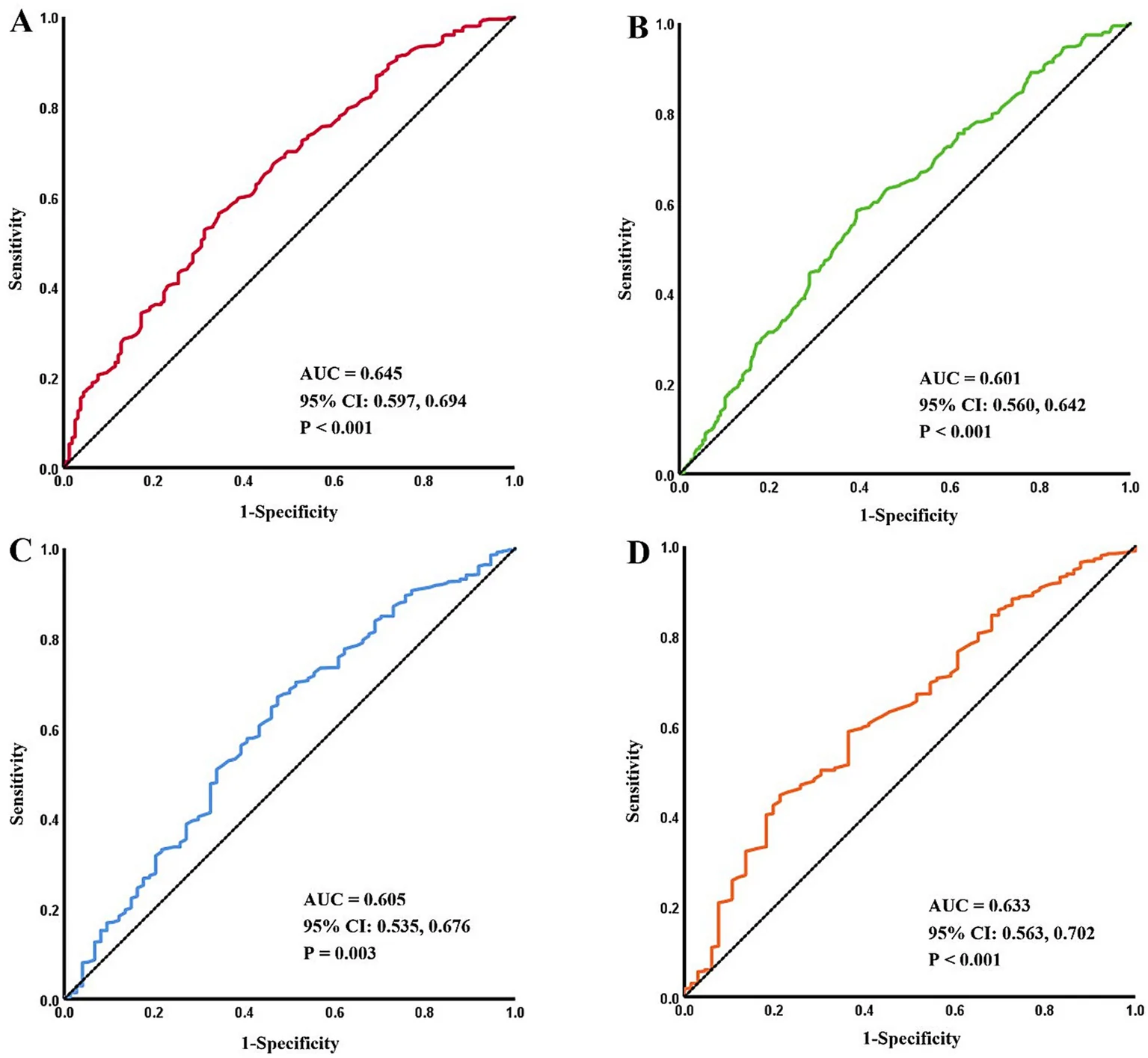
ROC curve evaluates the relationship between PNI and vomiting **(A)**, abdominal pain **(B)**, bloody stool **(C)**, and proteinuria **(D)**. ROC, receiver operating characteristic; AUC, area under the curve; CI, confidence interval; PNI, prognostic nutritional index.

As shown in Figure 2, ROC analysis demonstrated statistically significant associations between vomiting (AUC = 0.645, 95% CI: 0.597–0.694, *p* < 0.001), abdominal pain (AUC = 0.601, 95% CI: 0.560–0.642, *p* < 0.001), bloody stool (AUC = 0.605, 95% CI: 0.535–0.676, *p* = 0.003), and proteinuria (AUC = 0.633, 95% CI: 0.563–0.702, *p* < 0.001).

## 4 Discussion

The PNI, a composite metric derived from serum albumin levels and peripheral lymphocyte counts, has emerged as a robust biomarker for assessing systemic inflammation and nutritional status across various clinical conditions (14, 15). This real-world hospital-based study illustrates the novel application of PNI in pediatric IgAV, demonstrating that elevated PNI levels are independently associated with a reduced risk of gastrointestinal complications (such as vomiting, abdominal pain, and bloody stools) and renal manifestations (including proteinuria) after thorough adjustment for confounding variables. In future clinical practice, more attention should be paid to the PNI levels in children with IgAV to facilitate early identification of risk factors associated with gastrointestinal symptoms and renal injury.

Immunoglobulin A vasculitis, the most common systemic vasculitis in children, is characterized by IgA-mediated small-vessel inflammation affecting the skin, joints, GI tract, and kidneys (16, 17). GI bleeding is a life-threatening complication of IgAV. The NLR, as an indicator negatively correlated with PNI, can predict GI bleeding in adult IgAV patient (16). While pediatric-specific data are limited, the lymphocyte counts included in PNI may reflect similar immune dysregulation mechanisms. Malnutrition indicated by a low PNI can exacerbate intestinal barrier dysfunction, promote IgA deposition and mucosal inflammation (16, 18). In IgAV children requiring nutritional support, enriched formulas (such as alanyl-glutamine) reduced hospital stays and steroid dependence, underscoring the interplay between nutrition, inflammation, and outcomes (19). Additionally, Previous studies have shown that obesity is significantly associated with the severity and progression of IgAV (5, 6, 20). In children with chronic kidney disease (CKD), hypoalbuminemia (<35 g/L) and dyslipidemia—common in low-PNI groups—are associated with poor renal outcomes (21). PNI is also significantly correlated with SLE disease activity (22, 23). These findings align with data from lupus nephritis, where PNI ≤ 35.41 predicted ESRF, highlighting albumin’s role in maintaining endothelial integrity and modulating immune responses (24). A low PNI score also is a risk factor for AKI (25, 26). Minimal change disease patients with AKI demonstrated significantly lower serum albumin levels compared with those without AKI (27). In addition. Yu et al. (28) reported a lower PNI was associated with an increased risk of all-cause mortality in patients with renal failure. In this study, we also observed similar findings in children with IgAV, where lower PNI values were significantly associated with higher incidences of vomiting, abdominal pain, bloody stools, and proteinuria. The negative correlation observed in this study between PNI and clinical symptoms of IgAV may be attributed to hypoalbuminemia and lymphocytopenia. In IgAV, serum hypoalbuminemia may be associated with three conditions: malnutrition, proteinuria, and systemic small vessel inflammation. Hypoalbuminemia is known to be associated with malnutrition (29). Second, albumin is a negative acute-phase reactant, and its level may directly decrease with increasing inflammatory burden (30). Third, persistent proteinuria that may occur in IgAV patients can lead to hypoalbuminemia due to albumin loss. Furthermore, abnormal immune regulation of T cells during the acute phase of IgAV may result in a decreased peripheral blood lymphocyte count (31, 32). Although low PNI at disease diagnosis is associated with more gastrointestinal symptoms and kidney damage, the association may be bidirectional, and the causal relationship between them still requires further investigation.

The association between PNI and clinical symptoms of IgAV may stem from its dual reflection of nutritional deficiency and inflammatory activity. During the acute phase of IgAV, children often experience reduced nutritional intake and insufficient protein consumption due to vomiting and abdominal pain. Studies have shown that underweight related to malnutrition is an independent risk factor for the progression of kidney disease in IgAN (4). Additionally, hypoalbuminemia caused by cytokine-driven catabolism and oxidative stress is common in CKD (21, 24), and lower PNI correlates with worse renal outcomes (21). IgA is a systemic vasculitis characterized by inflammation that leads to lymphocyte apoptosis, downregulating lymphocyte differentiation and proliferation (29). On the other hand, lymphopenia may indicate T-cell exhaustion and impaired immune regulation, which may further promote the production of autoantibodies (30). Clinically, the concise characteristics of the PNI indicator facilitate routine monitoring and help implement early interventions for high-risk patients, such as those requiring nutritional optimization and immunosuppressive therapy.

Regarding the association between PNI levels at diagnosis and proteinuria rather than hematuria, we propose that proteinuria may reflect ongoing glomerular damage and is more closely related to the nutritional-inflammatory state indicated by PNI, whereas hematuria may be less associated with nutritional status. In IgAV, gastrointestinal involvement may be closely linked to systemic inflammation and mucosal immune dysregulation, thus the correlation between PNI and gastrointestinal symptoms is more pronounced than that with renal involvement. The observed associations between PNI and clinical variables in this study support this view. Although the causal relationship between PNI and clinical symptoms of IgAV requires further clarification, these findings suggest that clinicians should promptly recognize and monitor for potential symptoms in children with low PNI.

Although ROC analysis showed statistically significant associations between vomiting, abdominal pain, bloody stools, and proteinuria, the AUC values ranged from approximately 0.60 to 0.65. These values indicate moderate predictive ability. However, the main finding of this study is that the associations remained significant after multivariable adjustment, thereby validating the statistical robustness of the association. The true clinical value of PNI may lie in its combination with other indicators to form a multimodal model, which awaits validation in subsequent external datasets.

The clinical implications are substantial. Unlike costly or invasive diagnostic modalities, PNI leverages routine laboratory parameters, offering a cost-effective and readily deployable tool for risk stratification in resource-limited settings. For instance, in multivariable models, the PNI enhances the predictive ability for sepsis when used in conjunction with procalcitonin (PCT) (14); in KD, the PNI is associated with the severity of coronary artery lesions (32). Integrating the PNI into existing diagnostic frameworks can optimize treatment decisions, particularly in identifying high-risk patients who may benefit from intensified monitoring or early immunomodulatory therapy (32). In this study, we confirmed a significant association between PNI and complications of IgAV in children. By reflecting both nutritional and inflammatory status, PNI provides a comprehensive perspective for assessing disease severity, thereby addressing the limitations of traditional markers such as CRP or PCT (14, 33). Future research should explore mechanistic links between PNI and vascular permeability in inflammatory diseases, while pragmatic trials could assess whether PNI-guided interventions improve outcomes.

Although our data has yielded valuable findings, several limitations remain. First, as a single-center retrospective study, the results may lack representativeness and might not be generalizable to other ethnic groups or populations. Second, as an observational study without genetic association analysis, it cannot establish a causal relationship between PNI and complications in children with IgAV. Moreover, the multiple classifications and subgroup analyses involved in this study have certain limitations of multiple comparisons. Third, despite including multiple confounding variables, some important factors such as genetic predisposition, genetic, dietary, environmental, socioeconomic factors, and psychological factors might have been overlooked. Finally, since PNI was calculated based on serum albumin and lymphocyte count measured by a single examiner without dynamic monitoring or continuous management, it is impossible to determine the impact of PNI’s continuous exposure and dynamic evolution levels on complications in children with IgAV. In the future, we will adopt analyses that recommend the use of continuous modeling methods (such as restricted cubic splines), which may provide more meaningful dose–response relationships.

## 5 Conclusion

This study confirms that higher levels of PNI are significantly associated with a lower risk of complications (including vomiting, abdominal pain, bloody stool, and proteinuria) in pediatric IgAV, not only providing robust evidence for the inclusion of PNI in the clinical management of children with IgAV, but also expanding the research field of nutritional metabolism and IgAV. Future studies are recommended to conduct more large-scale prospective randomized clinical controlled trials, combined with artificial intelligence, bioinformatics, and cellular and animal experiments, to explore the potential associations between them.

## Data Availability

The original contributions presented in the study are included in the article/Supplementary material, further inquiries can be directed to the corresponding author.
