# Supplementary material for: Association between prognostic nutritional index and complications in children with immunoglobulin A vasculitis
Source: Front Nutr. 2026 Aug 17;13:1853071. doi: 10.3389/fnut.2026.1853071 (PMC13526555; doi:10.3389/fnut.2026.1853071)
Supplement: Supplementary file 1 [file Table_1.DOCX]

Supplemental Table 1. Sensitivity analysis: group by mean, tertiles, and quartiles of PNI

| Variables | Model 1 | | Model 2 | | Model 3 | |
| --- | --- | --- | --- | --- | --- | --- |
|  | OR (95% CI) | P value | OR (95% CI) | P value | OR (95% CI) | P value |
| **Vomiting** |  |  |  |  |  |  |
| PNI (Mean) |  |  |  |  |  |  |
| Low PNI | Ref |  | Ref |  | Ref |  |
| High PNI | 0.419 (0.288, 0.609) | <0.001 | 0.416 (0.278, 0.624) | <0.001 | 0.421 (0.280, 0.634) | <0.001 |
| PNI (Tertiles) |  |  |  |  |  |  |
| T1 | Ref |  | Ref |  | Ref |  |
| T2 | 0.551 (0.363, 0.837) | 0.005 | 0.574 (0.366, 0.900) | 0.016 | 0.538 (0.341, 0.849) | 0.008 |
| T3 | 0.366 (0.233, 0.576) | <0.001 | 0.344 (0.211, 0.560) | <0.001 | 0.351 (0.215, 0.575) | <0.001 |
| PNI (quartiles) |  |  |  |  |  |  |
| Q1 | Ref |  | Ref |  | Ref |  |
| Q2 | 0.654 (0.415, 1.032) | 0.068 | 0.638 (0.389, 1.046) | 0.075 | 0.609 (0.368, 1.006) | 0.053 |
| Q3 | 0.447 (0.274, 0.729) | 0.001 | 0.548 (0.323, 0.929) | 0.025 | 0.519 (0.305, 0.885) | 0.016 |
| Q4 | 0.262 (0.150, 0.457) | <0.001 | 0.218 (0.119, 0.399) | <0.001 | 0.221 (0.120, 0.405) | <0.001 |
| **Abdominal pain** |  |  |  |  |  |  |
| PNI (Mean) |  |  |  |  |  |  |
| Low PNI | Ref |  | Ref |  | Ref |  |
| High PNI | 0.491 (0.366, 0.660) | <0.001 | 0.436 (0.319, 0.598) | <0.001 | 0.408 (0.295, 0.565) | <0.001 |
| PNI (Tertiles) |  |  |  |  |  |  |
| T1 | Ref |  | Ref |  | Ref |  |
| T2 | 0.675 (0.471, 0.967) | 0.032 | 0.665 (0.455, 0.971) | 0.034 | 0.605 (0.409, 0.895) | 0.012 |
| T3 | 0.485 (0.338, 0.695) | <0.001 | 0.407 (0.277, 0.598) | <0.001 | 0.369 (0.246, 0.551) | <0.001 |
| PNI (quartiles) |  |  |  |  |  |  |
| Q1 | Ref |  | Ref |  | Ref |  |
| Q2 | 0.902 (0.594, 1.370) | 0.629 | 0.860 (0.554, 1.335) | 0.502 | 0.775 (0.491, 1.224) | 0.274 |
| Q3 | 0.502 (0.331, 0.761) | 0.001 | 0.497 (0.320, 0.772) | 0.002 | 0.434 (0.275, 0.685) | <0.001 |
| Q4 | 0.409 (0.268, 0.622) | <0.001 | 0.311 (0.197, 0.490) | <0.001 | 0.267 (0.166, 0.430) | <0.001 |
| **Bloody stool** |  |  |  |  |  |  |
| PNI (Mean) |  |  |  |  |  |  |
| Low PNI | Ref |  | Ref |  | Ref |  |
| High PNI | 0.529 (0.319, 0.877) | 0.014 | 0.536 (0.313, 0.916) | 0.023 | 0.506 (0.292, 0.877) | 0.015 |
| PNI (Tertiles) |  |  |  |  |  |  |
| T1 | Ref |  | Ref |  | Ref |  |
| T2 | 0.462 (0.255, 0.837) | 0.011 | 0.471 (0.251, 0.884) | 0.019 | 0.485 (0.254, 0.927) | 0.029 |
| T3 | 0.482 (0.268, 0.864) | 0.014 | 0.487 (0.262, 0.905) | 0.023 | 0.531 (0.276, 1.022) | 0.058 |
| PNI (quartiles) |  |  |  |  |  |  |
| Q1 | Ref |  | Ref |  | Ref |  |
| Q2 | 0.699 (0.363, 1.231) | 0.196 | 0.729 (0.379, 1.401) | 0.343 | 0.761 (0.386, 1.489) | 0.429 |
| Q3 | 0.389 (0.192, 0.789) | 0.009 | 0.472 (0.223, 1.001) | 0.050 | 0.454 (0.207, 0.996) | 0.049 |
| Q4 | 0.429 (0.215, 0.856) | 0.016 | 0.417 (0.201, 0.864) | 0.019 | 0.474 (0.220, 1.020) | 0.056 |
| **Proteinuria** |  |  |  |  |  |  |
| PNI (Mean) |  |  |  |  |  |  |
| Low PNI | Ref |  | Ref |  | Ref |  |
| High PNI | 0.448 (0.259, 0.774) | 0.004 | 0.453 (0.261,0.786) | 0.005 | 0.485 (0.278, 0.847) | 0.011 |
| PNI (Tertiles) |  |  |  |  |  |  |
| T1 | Ref |  | Ref |  | Ref |  |
| T2 | 0.807 (0.457, 1.426) | 0.461 | 0.774 (0.433, 1.385) | 0.389 | 0.768 (0.428, 1.378) | 0.376 |
| T3 | 0.376 (0.188, 0.753) | 0.006 | 0.384 (0.190, 0.775) | 0.008 | 0.404 (0.199, 0.819) | 0.012 |
| PNI (quartiles) |  |  |  |  |  |  |
| Q1 | Ref |  | Ref |  | Ref |  |
| Q2 | 0.676 (0.357, 1.280) | 0.229 | 0.604 (0.314, 1.162) | 0.131 | 0.589 (0.304, 1.140) | 0.116 |
| Q3 | 0.563 (0.288, 1.102) | 0.094 | 0.588 (0.296, 1.167) | 0.129 | 0.600 (0.301, 1.194) | 0.146 |
| Q4 | 0.254 (0.107, 0.600) | 0.002 | 0.254 (0.106, 0.606) | 0.002 | 0.266 (0.111, 0.638) | 0.003 |

For vomiting, Model 1 involved no covariates adjustment; Model 2 adjusted for WBC, SBP, ALT, UA, SCr, and APTT only; and Model 3 adjusted for WBC, SBP, ALT, UA, SCr, APTT, CRP, ESR, C4, IgA, and IgG.

For abdominal pain, Model 1 involved no covariates adjustment; Model 2 adjusted for WBC, HB, PLT, SCr, and APTT only; and Model 3 adjusted for WBC, HB, PLT, SCr, APTT, ASO, ESR, C4, IgA, and IgG.

For bloody stools, Model 1 involved no covariates adjustment; Model 2 adjusted for WBC, PLT, SBP, DBP, UA, and APTT only; and Model 3 adjusted for WBC, PLT, SBP, DBP, UA, APTT, CRP, ESR, IgG, and IgM.

For proteinuria, Model 1 involved unadjusted covariates; Model 2 adjusted for UA, urea nitrogen, and APTT only; and Model 3 adjusted for UA, urea nitrogen, APTT, and IgG.

WBC, white blood cell; SBP, systolic blood pressure; ALT, alanine aminotransferase; UA, uric acid; SCr, serum creatinine; APTT, activated partial thromboplastin time; CRP, C-reactive protein; ESR, erythrocyte sedimentation rate; C4, complement C4; IgA, immunoglobulin A; IgG, immunoglobulin G; IgM, immunoglobulin M; PLT, platelet count; DBP, diastolic blood pressure.
